# Global Identification of Small Ubiquitin-related Modifier (SUMO) Substrates Reveals Crosstalk between SUMOylation and Phosphorylation Promotes Cell Migration * [Fn FN2]

**DOI:** 10.1074/mcp.RA117.000014

**Published:** 2018-02-08

**Authors:** Ijeoma Uzoma, Jianfei Hu, Eric Cox, Shuli Xia, Jianying Zhou, Hee-Sool Rho, Catherine Guzzo, Corry Paul, Olutobi Ajala, C. Rory Goodwin, Junseop Jeong, Cedric Moore, Hui Zhang, Pamela Meluh, Seth Blackshaw, Michael Matunis, Jiang Qian, Heng Zhu

**Affiliations:** From the ‡Department of Pharmacology and Molecular Sciences, Johns Hopkins University School of Medicine, Baltimore, Maryland 21205;; §The Center for High-Throughput Biology, Johns Hopkins University School of Medicine, Baltimore, Maryland 21205;; ¶Department of Ophthalmology, Johns Hopkins University School of Medicine, Baltimore, Maryland 21205;; ‖Department of Neuroscience, Johns Hopkins University School of Medicine, Baltimore, Maryland 21205;; **Department of Neurology, Johns Hopkins University School of Medicine, Baltimore, Maryland 21205;; ‡‡Hugo W. Moser Research Institute at Kennedy Krieger, Baltimore, Maryland 21205;; §§Department of Pathology, Johns Hopkins University School of Medicine, Baltimore, Maryland 21205;; ¶¶Department of Biochemistry and Molecular Biology, Bloomberg School of Public Health, Johns Hopkins University, Baltimore, Maryland 21205

## Abstract

Proteomics studies have revealed that SUMOylation is a widely used post-translational modification (PTM) in eukaryotes. However, how SUMO E1/2/3 complexes use different SUMO isoforms and recognize substrates remains largely unknown. Using a human proteome microarray-based activity screen, we identified over 2500 proteins that undergo SUMO E3-dependent SUMOylation. We next constructed a SUMO isoform- and E3 ligase-dependent enzyme-substrate relationship network. Protein kinases were significantly enriched among SUMOylation substrates, suggesting crosstalk between phosphorylation and SUMOylation. Cell-based analyses of tyrosine kinase, PYK2, revealed that SUMOylation at four lysine residues promoted PYK2 autophosphorylation at tyrosine 402, which in turn enhanced its interaction with SRC and full activation of the SRC-PYK2 complex. SUMOylation on WT but not the 4KR mutant of PYK2 further elevated phosphorylation of the downstream components in the focal adhesion pathway, such as paxillin and Erk1/2, leading to significantly enhanced cell migration during wound healing. These studies illustrate how our SUMO E3 ligase-substrate network can be used to explore crosstalk between SUMOylation and other PTMs in many biological processes.

The construction of comprehensive networks linking protein substrates to their respective modifying enzymes is critical to increasing our functional understanding of the role of posttranslational modifications (PTMs)^1^ in signal transduction. Although many PTMs are carried out by individual enzymes (*e.g.* protein phosphorylation by protein kinases), some PTMs are regulated by complex enzymatic cascades (*e.g.* conjugation of small ubiquitin-related modifier (SUMO) to cellular proteins on lysine residues). SUMOylation is an essential PTM that controls a broad range of physiological processes, including DNA repair, transcriptional regulation, and nuclear import (1–8). The vertebrate genome encodes three distinct SUMO isoforms (SUMO1, SUMO2, and SUMO3), which are conjugated to substrate proteins via the SUMOylation enzymatic cascade. A single E1-activating enzyme (SAE1/SAE2 heterodimer) and E2-conjugating enzyme (Ubc9), and several E3 ligases mediate conjugation of SUMO to lysine residues on target proteins. Initially, it was unclear whether SUMO E3 ligases existed, because modification of many substrates did not require the presence of an E3 ligase *in vitro* (9). In contrast to the ubiquitylation cascade, which includes >600 E3 ligases, only ∼15 SUMO E3 ligases have been reported. These contrasts raise three key questions about the role of SUMO E3 ligases. First, do the E3 ligases determine global substrate specificity? Second, do individual SUMO E3 ligases show preference for SUMO isoforms? Finally, do individual SUMO E3 ligases selectively modify specific protein sub-families? Current techniques have not adequately provided answers to these questions.

Proteomic studies have identified thousands of SUMOylated human proteins conjugated to SUMO1 and SUMO2, by affinity purification of SUMO conjugates followed by mass spectrometry (10–13, 29, 43, 59). Although these studies have considerably increased the number of known SUMO substrates, the lack of connection to their upstream E3 ligases remains a roadblock to our understanding of how protein SUMOylation is dynamically regulated in mammalian cells. In this study, we developed an activity-based method for elucidating the global SUMO E3 ligase substrate network, by employing a human proteome microarray (HuProt™) containing >17,000 individually purified proteins (14). Utilizing *in vitro* array-based SUMOylation reactions with purified recombinant E1, E2, and E3s (*i.e.* PIAS1–4, RanBP2, and TOPORS), we systematically identified >1,700 E3 ligase-dependent substrates that are selectively modified with SUMO1 and/or SUMO2. Gene ontology analysis revealed a significant enrichment of protein kinases as SUMO substrates. *In cellulo* validation of members of the mitogen-activated protein kinase (MAPK) family identified an essential role for SUMOylation in kinase signaling. Further *in vivo* characterization of SUMO modification of a nonreceptor tyrosine kinase PYK2 demonstrated novel intramolecular crosstalk, where SUMOylation promotes cell migration via activation of PYK2.

## EXPERIMENTAL PROCEDURES

### 

#### 

##### Protein Purification

E3 ligase purification: Full length PIAS1, PIAS3, and PIAS3ΔSUMO were expressed in bacterial as glutathione *S*-transferase (GST) fusions in the pDEST15 bacterial expression vector. PIASxβ and PIASγ were expressed in bacteria in the pQLink 6XHis plasmid. Fragments of TOPORS (268–644) and the RanBP2 IR region were subcloned and expressed in pDEST15. All constructs were expressed in *Escherichia coli* and purified with Glutathione Sepharose 4B (GE Healthcare, Wauwatosa, WI) or Nickel NTA agarose (Qiagen, Germantown, MD).

##### In Vitro SUMOylation

E1 (standard 200 nm; low 35 nm), E2 (standard 600 nm; low 15 nm) were added to S^35^ radioactively labeled substrate (T_N_T Quick Coupled Transcription/Translation, Promega, Madison, WI). Assays performed with low concentrations of E1 and E2 were supplemented with E3 ligases (5–20 nm). Reaction mixtures were supplemented with energy mix buffer system (17). Reactions were incubated at 37 °C for 1 h, then quenched by addition of SDS-PAGE sample buffer and analyzed by SDS-PAGE and autoradiography.

##### Pilot Protein Microarray Fabrication

SUMO substrate open reading frames (ORFs) were expressed as GST fusion proteins in yeast. Cultures (6 ml) were grown at 30 °C to an optical density at 600 nm of 0.7 to 0.9 and induced with 2% galactose for 4 to 6 h. Harvested cells were lysed with glass beads in lysis buffer (100 mm Tris-HCl (pH 7.4), 100 mm NaCl, 1 mm EGTA, 0.1% 2-mercaptoethanol, 0.5 mm phenylmethylsulfonyl fluoride (PMSF), 0.1% Triton X-100 plus protease inhibitor mixture (Roche, Indianapolis, IN). GST fusion proteins were bound to glutathione beads (GE Healthcare) for 1 h at 4 °C and washed three times with wash buffer I (50 mm Tris-HCl, pH 7.4, 100 mm NaCl, 1 mm EGTA, 0.1% Triton X-100, 0.1% β-mercaptoethanol, and 0.5 mm PMSF) and three times with wash buffer II (50 mm HEPES, pH 7.4, 100 mm NaCl, 1 mm EGTA, 10% glycerol, 0.1% β-mercaptoethanol, and 0.5 mm PMSF) and eluted by glutathione competition elution buffer (100 mm Tris-HCl, pH 8.0, 100 mm NaCl, 10 mm MgCl_2_, 40 mm glutathione, and 30% glycerol). The eluate was collected through a filter unit and stored in a 384-well plate. Sixty-six SUMO substrate proteins were successfully purified, as determined by probing with anti-GST antibody. The purified substrates along with 16 recombinant control proteins, acquired through generous contributions, were printed in duplicate on modified glass (Full Moon Biosystems, Sunnyvale, CA) microscope slides using a 48-pin contact printer (Bio-Rad, Hercules, CA) employing four pins.

##### Protein Microarray SUMOylation

Protein chips were incubated overnight in BSA blocking buffer at 4 °C. SUMOylation on the protein chips was performed under two assays conditions. Chips were incubated with high concentrations of E1 (2.3 μm) and E2 (6.25 μm) and a positive control for control SUMOylation. To evaluate the activity of E3 ligases, limiting concentrations of E1 (45 nm) and E2 (125 nm) were supplemented with recombinant E3 ligases (5–20 nm). All mixtures included 0.7 μm mature SUMO-Alexa555, 5 mm ATP in 20 mm HEPES, pH 7.4, 100 mm NaCl, 10 mm MgCl_2_, 0.1 mm dithiothreitol. The SUMOylation reactions were carried out in a humidity chamber for 90 min at 37 °C then washed with TBST, followed by 1% SDS solution at 55 °C, rinsed with water and spun dry. Negative controls containing SUMO reaction mixture without enzymes were run in parallel. All conditions were performed in triplicate.

##### Data Analysis

To normalize the signal, we assume that the real reaction/signal are rare and almost evenly dispersed in each block, thus we force each block on a chip to have a median signal intensity of one. To be included as a positive hit, the duplicate spots of each gene must both have signal intensities (Foreground/Background ratio) five standard deviations above the mean. Additionally, positive hits from the triplicates of each enzymatic reaction must be identified by at least two of the three replicates. Positive hits also identified on negative control protein microarrays were removed. The names of all genes were checked and nonofficial gene name were replaced by official gene symbol name.

##### SUMOylation Substrate Network

We used Cytoscape to create the SUMOylation substrate network. The E3 ligase/SUMO pairing and substrates are represented by large filled circles and corresponding small filled circles, respectively. The E3 ligase and ligase-specific substrates are marked by same ligase/SUMO-specific color with substrates circled around the E3 ligase. For example, PIAS3/SUMO2 and its specific substrates are marked by blue. The shared substrates are marked by gray color and connected to corresponding case by case-specific color lines. For example, the substrate sets shared by PIAS3/SUMO2 and PIAS1/SUMO1 are connected to PIAS3/SUMO2 by green colored lines, and connected to PIAS1/SUMO1 by gold colored lines.

##### Phylogenetic Kinase Tree Overlaid with SUMOylation Enrichment

The amino acid sequences of kinase domain of all human kinase proteins have been annotated by Manning *et al.* with Hidden Markov Model (15). We collected these sequences from kinase.com and built the phylogenetic tree by Mega 5 (16). We marked different kinase family by distinct color shadow and marked the SUMOylated kinases with a red circle.

##### Kinase Protein-Protein and KSR Interaction Network

Seventy-one of the 2150 SUMOylation substrates (not including the substrates of high concentration E1 and E2) are kinases. To analyze the relationship of these 71 SUMOylation kinases, we built their functional relationship network. In this network, two kinases are connected by an undirected orange line if there is protein-protein interaction (PPI) between them. In addition, two kinases are connected by a directed green line with an arrow pointing to the substrate if there is a known kinase-substrate relationship between them. The kinases that do not have phosphorylation or PPI relationships are represented by orphan node.

##### Cell-based SUMOylation Assays

Candidate substrates were subcloned into the PCAGIG-V5 mammalian expression vector, SUMO1 and SUMO2 were subcloned into PCAGIG-MYC mammalian expression vector, and E3 ligases were subcloned into PSG5-FLAG mammalian expression vector. V5-substrate and MYC-SUMO were transfected together with and without FLAG-E3 ligases constructs with Fugene 6 transfection reagent (Promega) into HeLa cells seeded at 2E10^5^ cells per well. After 48 h, the cells were washed with phosphate buffered saline and lysed in RIPA buffer containing 20 mm *N*-ethylmaleimide and 1% SDS, to inhibit deSUMOylation and dissociate noncovalent protein complexes. Anti-V5-agarose beads were added (Sigma-Aldrich, St. Louis, MO) for 2 h to immunoprecipiate the substrate. Immunoprecipitates were resolved by SDS-PAGE and subject to immunoblotting to detect SUMOylation.

##### PYK2-SUMO1 Site Mapping

4E10^7^ HeLa cells were cotransfected with V5-PYK2, MYC-SUMO1, and FLAG-PIAS1 using Fugene 6 transfection reagent (Promega). After 48 h, cells were rinsed with warm PBS then lysed with RIPA buffer (50 mm Tris-HCl, pH 7.4, 150 mm NaCl, 2 mm EDTA, 1% Triton-X-100) containing 0.5 mm PMSF, Roche Protease Inhibitor Mixture, 20 mm *N*-ethylmaleimide and 1% SDS. The concentrated lysate was spun down to clear debris and the supernatant was diluted with RIPA without SDS, to a final concentration of 0.2% SDS. V5-PYK2 was immunoprecipitated with Anti-V5-agarose (Sigma Aldrich), washed three times with RIPA containing 0.1% SDS. PYK2 was eluted using V5 peptide (0.5 mg/ml). Eluate was incubated with anti-MYC agarose to isolate PYK2-SUMO1. Beads were again washed three times with RIPA containing 0.1% SDS. PYK2-SUMO1 was eluted from beads in 0.4 m ammonium bicarbonate and 8 m urea. TCA precipitation was used to concentrate the eluate and the pellet was resuspended in 8 m urea. The sample was heated to 100 °C for 10 min in LDS with β-mercaptoethanol, then fresh iodoacetamide was added to 50 mm concentration, the mixture was incubated in the dark at room temperature for 30 min. The sample was resolved by SDS -PAGE using a 12% NuPage Bis-Tris gel (Life Technologies, Carlsbad, CA) then silver stained with the SilverQuest kit (Life Technologies). Four bands corresponding to PYK2-SUMO1 were excised from the gel, destained, and subject to tryptic digestion (1:20 trypsin/substrate ratio). The resulting peptides were separated on a Dionex Ultimate 3000 RSLCnano system (Thermo Scientific, Wilmington, DE) with a 75 μm x 15 cm Acclaim PepMap100 separating column (Thermo Scientific) protected by a 2 cm guarding column (Thermo Scientific). Mobile phase consisted of 0.1% formic acid in water (A) and 0.1% formic acid 95% acetonitrile (B). The gradient profile was set as following: 4–30% B for 40 min, 30–45% B for 10 min, 45–95% B for 10 min. MS analysis was performed using an Orbitrap Velos Pro mass spectrometer (Thermo Scientific). The spray voltage was set at 2.2 kV. Spectra (AGC 1 × 10^6^) were collected from 400–1800 m/z at a resolution of 60,000 followed by data-dependent HCD MS/MS (at a resolution of 7500, collision energy 35%, activation time 0.1 ms) of the 10 most abundant ions. MS/MS spectra were searched against a human IPI reference database (V3.87) using the SEQUEST engine in Proteome Discover 1.3. Searching parameters included mass tolerance of precursor ions (± 20 ppm) and product ion (± 0.06 Da), dynamic modification of carboxyamidomethylated Cys (+ 57.0215 Da), dynamic mass shifts for oxidized Met (+ 15.9949 Da), and dynamic modification of SUMO1 C-terminal peptide (ELGMEEEDVIEVYQEQTGG) or target peptide terminal attached to the modified K. Only b and y ions were considered during the database match.

##### PYK2 In Vitro Kinase Assay

GST-PYK2 WT was purified from yeast using glutathione Sepharose 4B (GE Healthcare). Following washes PYK2 was left on beads and separated into two aliquots. One aliquot was SUMOylated under standard conditions (reaction 1) with 90 nm E1, 300 nm E2, 5 mm ATP in 50 mm HEPES, pH 7.4, 100 mm NaCl, 10 mm MgCl_2_, and 0.1 mm dithiothreitol for 1 h at 37 °C with gentle shaking. The reaction mixture was removed, the beads were washed two times with SUMOylation buffer without enzymes or ATP, a final wash was performed with kinase buffer lacking ATP. The second aliquot served as a control (reaction 2) and was incubated with SUMOylation reaction buffer lacking enzymes, SUMO, and ATP. In the second phase of this reaction, the beads were again divided in two, creating four reaction conditions. Kinase buffer (50 mm HEPES, pH 7.4, 10 mm MgCl_2_, 10 mm MnCl_2_, 300 mm KCl, and 0.5% Nonidet P-40) with 1 mm cold ATP was added to one aliquot that had previously undergone SUMOylation (reaction 1.1) and one aliquot that had only been incubated with buffer (reaction 2.1). The paired aliquots were incubated with kinase buffer lacking ATP (reaction 1.2 and 2.2). The autophosphorylation and control reactions were allowed to proceed for 1 h at 30 °C with gentle shaking then all reactions were washed three times with kinase buffer lacking ATP. The beads were heated in 2X LDS sample buffer containing β-mercaptoethanol at 100 °C for 10 min, then resolved by SDS-PAGE and immunoblotted with anti-GST (Millipore), anti-SUMO1 (21C7) (Matunis laboratory), and anti-PYK2 pTyr402 (Life Technologies) antibodies.

##### SUMO Dependent PYK2-SRC Interaction

V5-tagged WT PYK2, 4KR PYK2, and PYK2 Tyr402F were all transfected with and without MYC-SUMO1 in HeLa cells. V5-tagged WT PYK2 and 4KR PYK2 were also cotransfected with flag-PIAS1 and MYC-SUMO1. 4KR PYK2 and Tyr402F were generated using the QuikChange II site directed mutagenesis kit (Agilent Technologies, Wilmington, DE). Cells were rinsed with warm phosphate buffered saline 48 h after transfection and lysed with Kamiya buffer (50 mm Tris-HCl, pH 7.4, 150 mm NaCl, 5 mm MgCl_2_, 1 mm EDTA, 1% Triton-X-100). The lysate was incubated with anti-V5-agarose for two hours. Beads were washed three times with TBST then boiled with 2× LDS containing β-mercaptoethanol. The immunoprecipitates were resolved by SDS-PAGE followed by immunoblotting with antibodies to SRC (Cell Signaling, Danvers, MA) and paxillin (Santa Cruz, Dallas, TX). Phosphospecific antibodies for paxillin pTyr118 and SRC pTyr416 (Cell Signaling) were used to assess phosphorylation status.

##### 2D Cell Scratch Assay

MDA-MB-231 cells were plated in 6 well format and infected with adenovirus constructs for WT PYK2, 4KR PYK2, and SUMO1 (generated using ViraPower™ Adenoviral Gateway™ Expression Kit, Life Technologies). When cells were confluent (24 h post infection), a scratch was made using a fine pipette tip and cells were gently washed three times with room temperature phosphate buffered saline. Cells were then placed in low serum (0.1% FBS) medium for 24 h. Phase contrast images of the same fields were taken at 0 h and 24 h following the scratch and cells migrating beyond the wound's edge were manually counted (*n* = 3 per condition).

##### Immunofluorescence Staining

Cells were plated in a glass slide chamber (Thermo Fisher, Westminster, MD) and infected with the designated adenoviral constructs. A scratch was made as previously described (2D scratch assay) and cells were placed in low serum medium for 4 h followed by fixation with 4% formaldehyde. Cells were permeabilized with 0.5% Triton-X-100 and blocked with normal goat serum. Cells were probed with anti-phospho-paxillin (Cell Signaling) followed by Cy3-conjugated goat-anti-rabbit 2^nd^ antibody. Following PBS washes, slides were counter stained with DAPI and fluorescence images were taken using Axivision apotome microscopy (Zeiss, Peabody, MA) with the same exposure time.

## RESULTS

### 

#### 

##### Assay Development for SUMO E3-Dependent Substrate Identification

To identify SUMO E3 ligase-dependent substrates, we employed an *in vitro* assay using the human proteome microarray (*i.e.* HuProt™), in combination with bioinformatics analysis to determine the enzyme-substrate relationships. First, we fabricated a pilot protein microarray containing multiple known SUMO substrates expressed and purified from bacteria, in order to optimize the reaction conditions for detecting SUMO E3 ligase-dependent substrates. The pilot microarray contained 60 previously identified substrates, SUMO1–4, E1 and E2 enzymes involved in SUMOylation, and GST and BSA as negative controls. To verify detectable substrate SUMOylation, we incubated the pre-blocked pilot microarray with high concentrations of SAE1/UBA2 (*i.e.* E1; 2.3 nm), Ubc9 (*i.e.* E2; 6.25 nm), in the standard SUMOylation reaction buffer in the presence of fluorescently labeled SUMO1 or SUMO2. Following incubation with this enzyme mixture, the pilot microarray was washed under denaturing conditions, and signals from covalently SUMOylated substrates were detected via fluorescence imaging. Under this condition of high E1/E2 (defined as 50X in the rest of the text; see Experimental Procedures for more details) concentrations, we observed significant SUMOylation signals from 41% of the known substrates (supplemental Fig. S1), consistent with previous observations that E3s are not required for *in vitro* SUMOylation of many substrates when carried out in the presence of high E1 and E2 concentrations (9, 17, 18). To determine the minimal detectable SUMOylation signal induced by E1 and E2 alone, we repeated SUMOylation reactions on our pilot protein microarrays using a dilution series of E1 and E2 enzymes until SUMOylation signals could be barely detected and therefore, defined as 1X E1/E2 condition (supplemental Fig. S1).

In parallel, we purified the 11 reported SUMO E3 ligases, including PIAS1–4, RanBP2, TOPORS, MAPL, Pc2, Rhes, HDAC, and HSP27, as recombinant proteins and tested their E3 ligase activity against their reported substrates under standard *in vitro* conditions (17). Six of the eleven tested E3 ligases - namely PIAS1–4, RanBP2, and TOPORS - showed robust SUMOylation activity to their previously reported substrates and were thus selected for further studies using protein microarrays (supplemental Fig. S2).

**Fig. 1.**
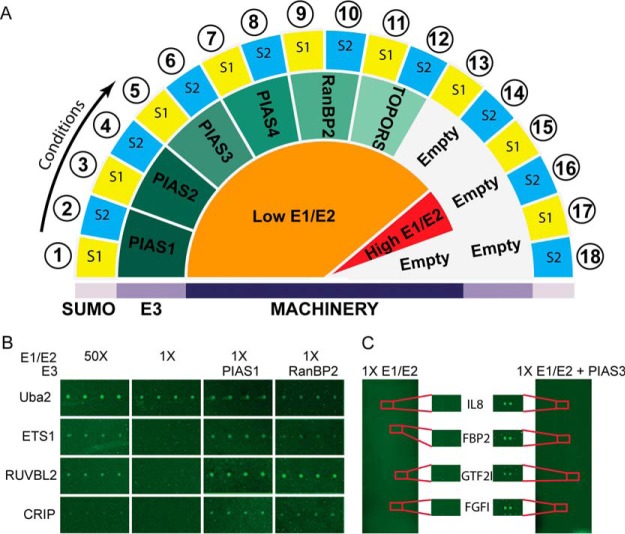
**Global Identification of SUMO E3 ligase-dependent substrates using the HuProt™ arrays.** *A*, Schematic describing the global SUMOylation assay design. SUMOylation reactions with high and low concentrations of recombinant E1/E2, paired with SUMO1 or SUMO2, were performed as controls. E3 ligases were spiked into the low E1/E2 conditions and performed with SUMO1 and SUMO2. All experimental and control conditions were performed in triplicate. *B*, E3 ligase activity was tested in pilot study performed on a specialized SUMOylation protein chip containing 82 control proteins. Low concentrations of E1/E2 allow for direct detection of E3 ligase activity compared with high E1/E2 controls reactions. *C*, PIAS3 SUMO2 substrates from the HuProt™ microarray are highlighted.

To identify E3 ligase-dependent substrates, we supplemented the reaction mixture (containing 1× E1/E2 enzymes) with active E3 ligases to conduct the SUMOylation reactions on the pilot array (Fig. 1*A*). We detected SUMO modification signals in all substrates previously identified under the 50X E1/E2 condition. Importantly, we also identified additional E3 ligase-dependent SUMOylation signals that could not be detected under low E1/E2 conditions. For certain substrates, such as CRIP, we detected robust SUMOylation signals in the presence of the E3 ligase RanBP2, even though these substrates were not found to be SUMOylated under high E1/E2 conditions (Fig. 1*B*). For the remainder of our experiments, the minimal amounts of E1 and E2 enzymes required for detecting E3-dependent substrates on protein microarrays were defined as 45 nm E1 and 12.5 nm E2 (referred to as 1× E1/E2).

##### Global Profiling of SUMO E3 Ligase-Substrate Relationships

We employed the HuProt™ array (14), comprised of ∼17,000 unique proteins, to systematically identify potential E3 ligase-dependent SUMO substrates in the human proteome. The optimized 1X E1/E2 assay condition was supplemented with the six E3 ligases and either SUMO1 or SUMO2, and then applied to the HuProt™ array (Fig. 1*C*). HuProt™ arrays incubated with 50X E1/E2 paired with SUMO1 or SUMO2 were used as a SUMOylation positive control. Conversely, 1× E1/E2 paired with either SUMO1 or SUMO2 were incubated on the HuProt™ array as a negative control.

**Fig. 2.**
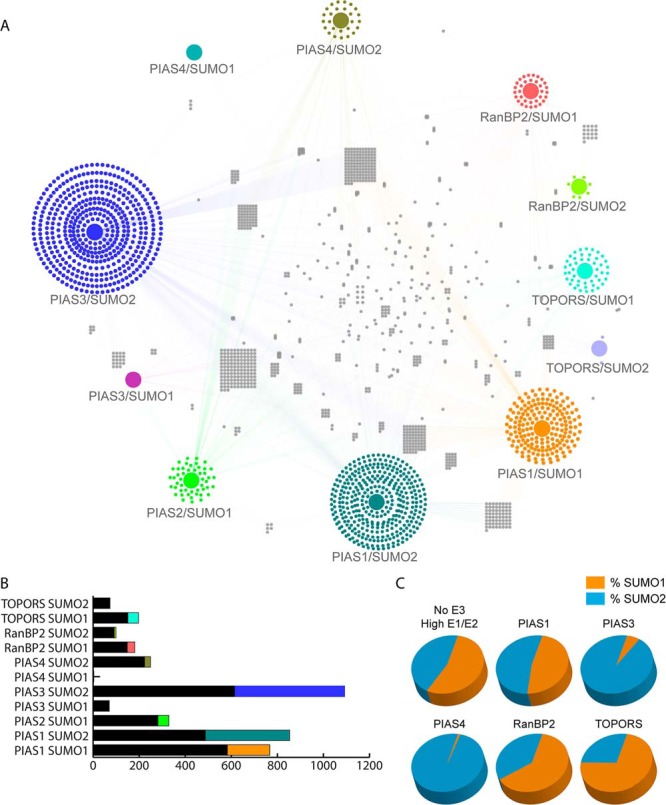
**Specificity in E3 ligase mediated-SUMOylation.** *A*, A network showing the connections between each E3 ligase/SUMO isoform pairing and the modified substrates was generated using Cytoscape. The colored edges depict the connection to an upstream E3 ligase. Many substrates are connected to more than one E3 ligase/SUMO pairing, thus revealing the overlap and redundancy between E3 ligases. *B*, The proportions of substrates shared by E3 ligases as compared with the unique substrates targeted by each E3 ligase/SUMO pairing. The colored fractions represent the number of substrates unique to the reaction, whereas the black fractions represent the targets modified in at least two reactions. *C*, Preferential modification with SUMO1 *versus* SUMO2. The orange fraction represents the percentage of total hits modified with SUMO1; the blue fraction represents substrates modified with SUMO2. Substrates modified with SUMO1 and SUMO2 are counted in both fractions.

To ensure reproducibility of the assay, each SUMOylation reaction was performed in triplicate. A total of eighteen SUMOylation reactions were performed on 54 HuProt™ arrays under the conditions specified in Fig. 1*A* (Data set 1). Only those proteins that were found SUMOylated in at least two of the triplicate assays were recognized as reproducible hits. Under the 50× E1/E2 reaction condition, we detected 2346 and 1933 SUMOylated proteins in the presence of SUMO1 and SUMO2, respectively. Unfortunately, despite numerous attempts, the PIAS2-SUMO2 reactions yielded saturated images that could not be analyzed by the image acquisition software and therefore PIAS2-SUMO2 data is not incorporated in our data set. Under the 1X E1/E2 concentrations, addition of the six E3 ligases yielded a variable number of modified substrates, ranging from 3 to 1092 (Data set 3) (Fig. 2*A*). For example, the PIAS3-SUMO2 reactions identified 478 unique targets; whereas TOPORS-SUMO2 only modified two proteins specifically (Fig. 2*B*; Table I). Except for two reactions (PIAS3/SUMO1 and PIAS4/SUMO1), all assays identified specific targets. Collectively, a total of 2149 substrates were SUMOylated by all E3 ligases tested (Table I). By removing the hits in the 50× E1/E2 experiment from those obtained in the E3 ligase reactions under 1× E1/E2 condition, we revealed the E3 ligase-dependent targets. The known SUMOylation consensus motif occurred in 27.27–54.61% (M3 algorithm) or 20.2–56.62% (GPS-SUMO) (19) (depending on the E3 ligase) of the proteins modified in our assays (Table I) (Data set 2). We also observed that PIAS E3 ligase reactions targeted substrates with proportionally higher numbers of ZINC finger motifs than reactions lacking E3 ligases, or RanBP2 and TOPORS reactions (Table I).

**Table I:** 

	Total	Unique	Isoform Preference	SUMO sites M3	SUMO sites GPS SUMO	SIMs	Phospho-protein	Kinases	ZINC fingers
50X E1/E2 S1	2346	—	54.8%	799	844	240	1286	81	80
50X E1/E2 S2	1933	—	45.2%	687	715	205	1073	72	62
PIAS1 SUMO1	767	184	47.3%	293	334	105	445	32	39
PIAS1 SUMO2	853	366	52.7%	329	330	99	478	34	43
PIAS2 SUMO1	329	48	—	156	169	44	224	12	15
PIAS3 SUMO1	70	0	6%	26	31	9	47	4	4
PIAS3 SUMO2	1092	470	94%	413	402	136	601	32	55
PIAS4 SUMO1	3	0	1%	0	3	1	3	0	0
PIAS4 SUMO2	249	26	99%	136	141	37	181	10	11
RANBP2 SUMO1	181	33	62.8%	53	43	17	116	7	2
RANBP2 SUMO2	99	7	37.2%	27	20	7	51	4	1
TOPORS SUMO1	197	46	73%	60	63	15	111	6	10
TOPORS SUMO2	73	2	27%	25	28	8	50	7	3

##### Global Properties of SUMO E3-Substrate Relationship and Specificity

The possibility that individual SUMO E3 ligases target common substrates has been raised repeatedly throughout the literature; however, this remains largely unresolved because of inadequate characterization of global E3 ligase-substrate relationships. We used our E3 ligase-dependent SUMOylation data sets to construct a network depicting the substrate specificity of each SUMO E3 ligase tested. As illustrated in Fig. 2*A*, identified SUMO substrate proteins are connected to the E3 ligases by which they are modified. E3 ligases show variable degrees of overlap among the substrates of virtually all the pairwise E3 ligase comparisons. For example, PIAS4-SUMO2 and PIAS2-SUMO1 share 45.2% of their substrates, PIAS1-SUMO1 and PIAS2-SUMO1 share 13.4% of their substrates, and RanBP2-SUMO2 and PIAS3-SUMO2 share only 3.7% of their substrates. This observation suggests that, although some proteins are targeted by multiple different E3 ligases, there is also a great deal of specificity within the SUMOylation machinery. Indeed, >1000 substrates identified in our assays only occurred in one unique reaction out of the 18 that were carried out. Furthermore, we also observed that certain E3 ligases demonstrated a preference for a SUMO isoform. Under high concentrations of E1 and E2 we did not observe a bias for SUMO1 or SUMO2; however, under 1X E1/E2 conditions, the addition of E3 ligases shifted the balance. RanBP2 and TOPORS showed a preference for SUMO1 at a level of 62.8 and 73%, respectively; whereas PIAS3 and PIAS4 strongly favored modification with SUMO2 (94 and 99%). PIAS1 modified substrates equally with SUMO1 and SUMO2 (Table I; Fig. 2*C*). These observations suggest that certain SUMO E3 ligases play an important role not only in defining substrate specificity, but also in selecting a SUMO isoform for the modification.

**Fig. 3.**
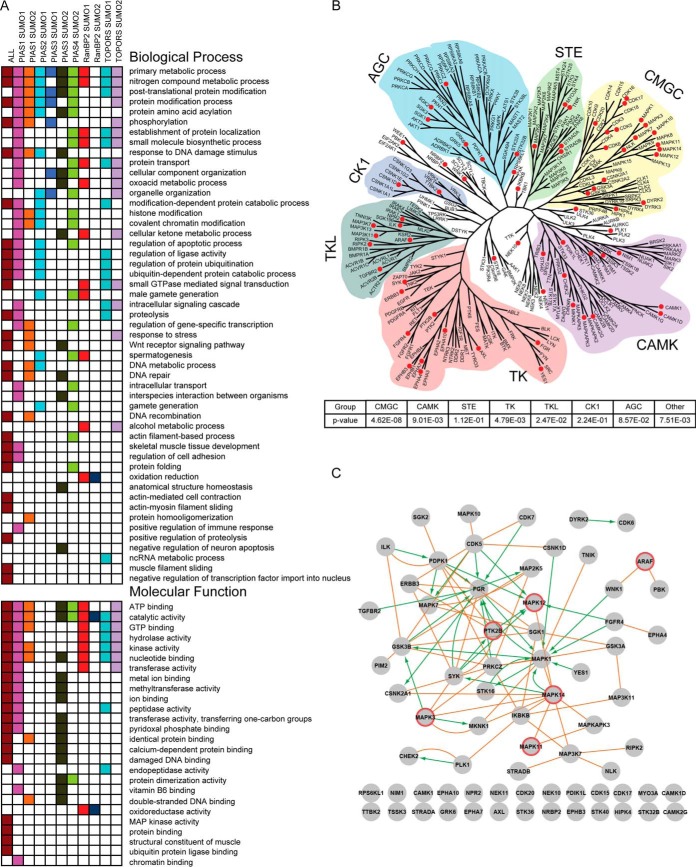
**Biological Classification of SUMOylated proteins.** *A*, Gene Ontology analysis of significant biological processes and molecular functions. Overrepresented (*p* < 0.05) biological process and molecular function GO categories were identified. The composite data set and each E3 ligase/SUMO pairing were analyzed separately. *B*, Phylogenetic kinase tree overlaid with SUMOylation enrichment the amino acid sequences of kinase domain of all human kinase proteins were collected to build the phylogenetic tree with Mega 5, a program designed to construct phylogenetic trees from aligned sequences. Kinase families are designated by distinct color and SUMOylated kinases are indicated by red circles. *C*, Kinase SUMOylation substrates in PPI and KSR network. Protein-protein interaction analysis was performed on the subset of kinases identified as SUMO substrates in our array-based assays (orange edges). Kinase substrate relationships (KSRs) are represented by green directional arrows. Kinases encircled in red were selected for validation studies.

To gain a deeper insight into the biologically relevant functions that SUMOylation and individual E3 ligases may regulate, we performed gene ontology analysis (GO) of our data set to discover statistically enriched substrates associated with biological processes, molecular functions, and cellular compartment (20). GO analysis was performed on the collection of all SUMO substrates, as well as each individual E3-SUMO pairing substrate set (Fig. 3*A* and supplemental Fig. S4*A*). The full set of proteins printed on the HuProt™ array served as the background for statistical analysis. Among the identified functional categories, we readily recovered several of the previously described GO terms associated with SUMOylation, such as response to stress, DNA damage, DNA recombination, protein localization, and protein transport, as well as enrichment in the nuclear compartment (2, 4, 6, 12, 21–23). Interestingly, we found many enriched molecular function categories that were related to enzymatic functions, such as kinase activity (24), MAP kinase activity, GTP hydrolysis activity, methyltransferase activity, and peptidase activity, which were not previously recognized as preferentially undergoing SUMOylation (Fig. 3*A*).

##### Protein Kinases Are Preferred SUMO Substrates

Although protein phosphorylation and dephosphorylation, catalyzed respectively by protein kinases and phosphatases, are shown to play a central role in regulating signal transduction, cross-talk between (de)phosphorylation and other types of PTMs can also directly regulate signal transduction. Multiple studies demonstrate that kinase activity and/or substrate specificity are subject to acetylation, ubiquitylation, and *O*-glycosylation (25–28). However, no systematic efforts have been reported that investigate crosstalk between SUMOylation and phosphorylation in eukaryotes. Surprisingly, we observed that protein kinases were significantly enriched among SUMOylation substrates (110 kinases identified; *p* < 9.76E-03). More specifically, these kinases were mostly modified by PIAS1-SUMO1/SUMO2, RanBP2-SUMO1, and TOPORS-SUMO1/SUMO2 combinations.

To globally examine the interplay between SUMOylation and phosphorylation, we annotated SUMOylated kinases on a kinase dendrogram, consisting of the 340 kinases printed on the HuProt™ array. We examined whether kinase groups are preferred SUMOylation substrates by calculating the significance of the enrichment (Fig. 3*B*). Though kinases in each of the seven major groups were found to be SUMOylated in our screen, the CMGC (*p* < 4.62E-08), TK (*p* < 4.79E-03), CAMK (*p* < 9.01E-03), and TKL (*p* < 2.47E-02) groups showed significant enrichment. For example, within the CMGC group most of the MAP kinases were found to be SUMOylated, including MAPK1, MAPK3, MAPK7, MAPK11, MAPK12, and MAPK14. Enrichment of the MAPK family was also reported in the largest mass spectrometry proteomics study which shares the greatest overlap with our data set (29).

To identify regulatory relationships among the SUMOylated kinases, we constructed an integrated network comprised of the PPI and kinase substrate relationships (KSR) (Fig. 3*C*) (30) (60). Of the 71 kinases that could be incorporated into this network, 46 kinases formed a tightly connected subnetwork, suggesting that multiple components in separate kinase signaling cascades might undergo SUMOylation. One interesting feature of this network is that MAPKs (*e.g.* MAPK1/Erk2, MAPK3/Erk1, MAPK12/p38γ, MAPK14/p38α)) and tyrosine kinases (*e.g.* PTK2B/PYK2, Fgr, Syk, ERBB3/Her3) are highly connected with each other and other kinases, suggesting potential cross-talk between phosphorylation and SUMOylation.

Our analysis revealed that many proteins that undergo phosphorylation were also identified as SUMOylation substrates. Furthermore, statistically significant overlap of substrates identified as SUMOylated proteins (this study) were also identified as kinase substrates undergoing phosphorylation in our prior study (*p* < 9.9E-14) (30). Even when kinases were excluded from these data sets, 1143 of phosphoproteins were also found to be SUMOylated (*p* < 1.38E-12).

##### Many MAPKs are SUMOylated in Cells

**Fig. 4.**
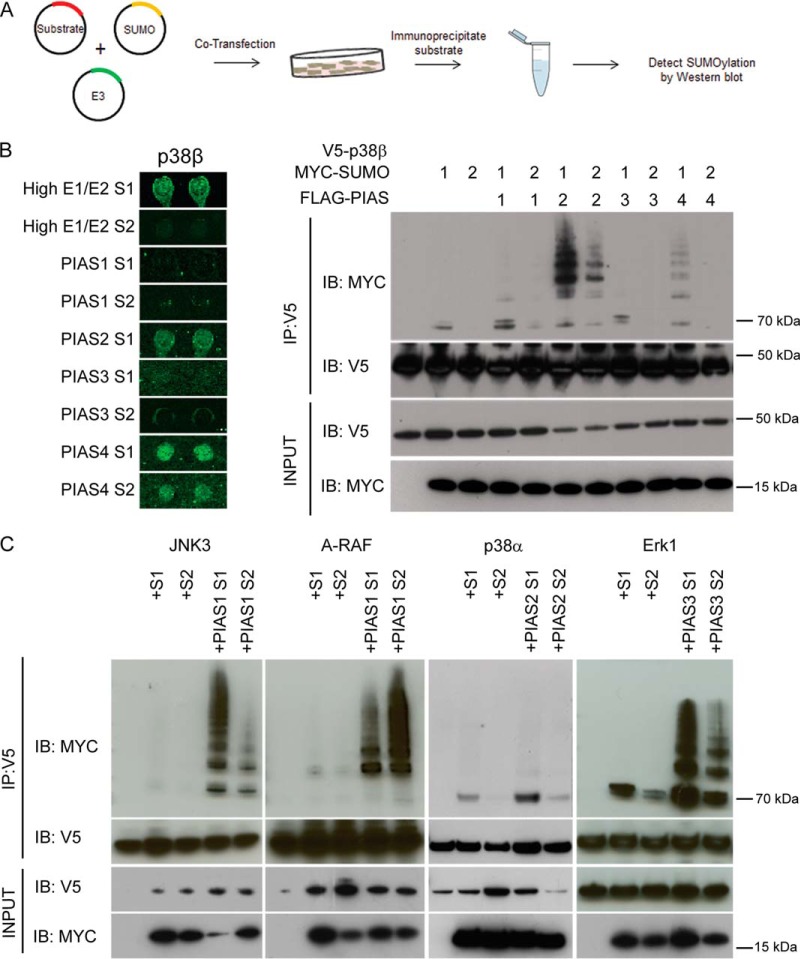
**Validation of E3 ligase specificity of in the MAPK kinase family.** *A*, Flow chart for validation studies. V5-tagged kinases were transfected into HeLa cells. In parallel each kinase is cotransfected with SUMO1 or SUMO2, and separately cotransfected with SUMO1 or SUMO2 and the indicated E3 ligase. V5-tagged substrates were immunoprecipitated, resolved by SDS-PAGE, then blotted with anti-MYC to detect SUMOylation. *B*, The fidelity of HuProt™ array E3 ligase results were tested both for the substrate and SUMO isoform specificity. *C*, Activity of PIAS E3 ligases was confirmed with four additional MAPK kinase substrates.

We elected to use SUMO1 and SUMO2 expression constructs to stimulate SUMOylation *versus* stress conditions because each candidate substrate may require a unique stress condition to drive the modification. With this approach, we were able to verify the *in vitro* microarray array data in a cellular setting, providing evidence that the targets in our data set are likely to be authentic. Given our above observations, we selected five MAPKs, namely ERK1, JNK3, p38β, p38α, and A-RAF, to determine whether these substrates are able to be SUMOylated by a particular combination of the SUMOylation machinery in transfected cells as observed in the HuProt™ array reactions. The selected kinases are highly connected in our network analysis and are targeted by a variety of E3 ligases. Each kinase ORF was fused to a V5 epitope in a mammalian expression vector, followed by cotransfection separately with SUMO1 and SUMO2 expression constructs. Designated E3 ligase constructs were also cotransfected to validate the fidelity of the array results in a cell-based system (Fig. 4*A*).

According to the HuProt™ array results, p38β was readily SUMOylated under five conditions, including 50× E1/E2 with SUMO1 and SUMO2, PIAS2-SUMO1, and PIAS4-SUMO1/SUMO2. To systematically validate our microarray results, we cotransfected HeLa cells with all E3 and SUMO isoform combinations that were tested using the *in vitro* HuProt™ arrays (Fig. 4*A*). SUMOylation was confirmed by immunoprecipitating (IP) the substrate proteins under denaturing conditions, followed by immunoblotting for SUMO modification (Fig. 4*A*). In addition, SUMO1 or SUMO2 alone was cotransfected with p38β. As a negative control, the p38β construct was transfected alone. Using an IP-coupled immunoblot (IB) analysis, p38β was confirmed to be SUMOylated under all expected conditions, with the exception of 50X E1/E2 SUMO1 and PIAS4-SUMO2. Consistent with our microarray analysis, in our cell-based experiment, p38β was modified by SUMO1 in the absence of an E3 ligase. Cotransfection with either PIAS1 or PIAS3 did not further enhance SUMOylation levels of p38β. Moreover, cotransfection of PIAS2 or PIAS4 strongly enhanced SUMOylation with SUMO1 as predicted by the protein microarray results (Fig. 4*B*). To further validate our microarray results, we selected four additional MAPKs for cell-based studies using conditions predicted by the protein mircoarrays. JNK3/MAPK10, A-RAF, p38α, and ERK1 were modified under conditions identical to our microarray analyses (Fig. 4*C*). These validation studies indicate that our microarray results can be largely reproduced for both E3 ligase activity and SUMO isoform specificity.

##### Crosstalk Between SUMOylation and Tyrosine Phosphorylation via PYK2

**Fig. 5.**
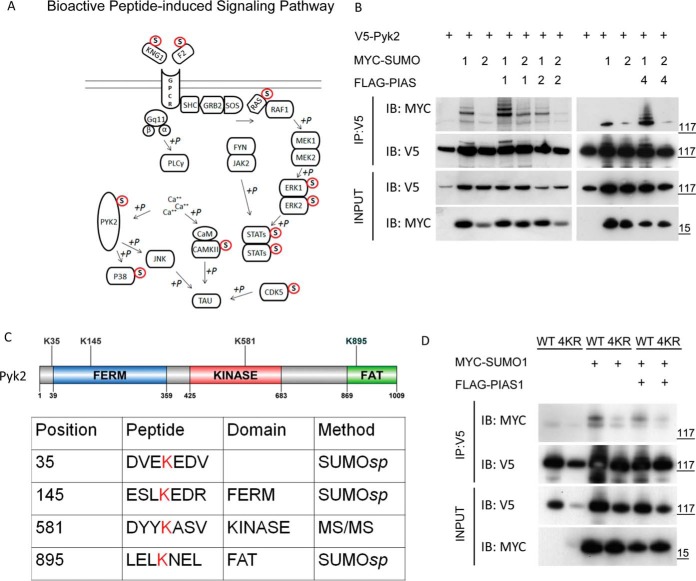
**PYK2 is a SUMOylated tyrosine kinase.** *A*, Bioactive peptide induced signaling pathway is enriched for SUMOylation substrates (*p* < 1.637 E10–4). Proteins that were recovered in our global SUMOylation assay are marked as SUMO modified. *B*, Validation of PYK2 SUMOylation and E3 ligase specificity in HeLa cells as indicated in Fig. 4*A*. SUMOylation of nonreceptor tyrosine kinase PYK2 was performed according to the conditions indicated in the global SUMOylation screen. *C*, Schematic representation of candidate attachment site lysines in PYK2. *D*, SUMOylation assay using PYK2 containing the four candidate lysine to arginine mutations (Fig 5*C*) demonstrated impaired SUMOylation in the 4KR mutant.

A surprising observation was that many cytosolic nonreceptor tyrosine kinases were SUMOylated in our protein microarray analysis, although SUMOylation has been commonly linked to nuclear components. Pathway analysis revealed several tyrosine kinase signaling pathways that were significantly enriched for SUMOylated proteins. An interesting example is the bioactive peptide-induced signaling pathway in which 11 of 26 components are found to be SUMOylated (*p* < 1.6E-03) (Fig. 5*A*). A nonreceptor tyrosine kinase PYK2 (also known as PTK2B, RAFTK, FAK2, CAK-β, or CADTK), which is known to play a central role in this signaling pathway, was SUMOylated by PIAS2 and PIAS4 in our microarray assays. PYK2 is a member of the focal adhesion kinase (FAK) family, which consists of FAK and PYK2. Focal adhesion kinases are critical activators of signal transduction pathways that regulate cell migration (31–33), proliferation (34), and survival (35). Focal adhesion kinases are also a point of convergence for signaling initiated by growth factors and G-protein coupled receptors, integrating information to regulate cell growth and migration via downstream MAPK signaling (Fig. 5*A*). Although cross-talk between SUMOylation and cytosolic tyrosine phosphorylation has not been reported, multiple downstream components of this pathway, such as the MAPKs, have been identified and validated as SUMOylated substrates in cells. Therefore, we focused on elucidating the molecular mechanism underlying SUMOylation and PYK2 phosphorylation in the remainder of our study.

##### Site-specific PYK2 SUMOylation

To confirm that PYK2 is SUMOylated in cells, a similar cell-based validation assay was performed. Cotransfection of *PYK2* and *SUMO1/2* expression constructs in the absence of the E3 ligases resulted in robust SUMOylation of PYK2 with SUMO1, but much less efficient conjugation with SUMO2 (Fig. 5*B*). The addition of PIAS1 or PIAS4 (*i.e.* PIASγ) in the cotransfection assays greatly enhanced the modification (*i.e.* laddering pattern) of PYK2 with SUMO1, indicating the presence of multiple SUMOylation events. On the other hand, cotransfection of SUMO2 with all three E3 ligases only resulted in moderate PYK2 SUMOylation. Conversely, PIAS2 (*i.e.* PIASβ) did not show detectable activity with either SUMO1 or SUMO2 (Fig. 5*B*). Based on these results, we analyzed the impact of SUMOylation on PYK2 activity following PIAS1-dependent SUMO1 modification.

To determine how SUMOylation regulates the function of PYK2, we first mapped SUMOylation sites on PYK2. We employed SUMO*sp* software to predict lysine residues with high probability of undergoing SUMOylation, and then targeted these for mutagenesis studies. The top three lysines identified were K35 adjacent to the FERM domain, K145 within the FERM domain, and K895 (a strong consensus site) within the FAT domain. None of these sites were in the kinase domain (Fig. 5*C*). To determine whether these lysine residues were SUMOylated *in cellulo*, we generated a triple mutant (3KR) combining all three lysine-to-arginine mutations. Relative to WT PYK2, the major SUMOylated species (lowest molecular weight band) remained present in the 3KR mutant, although the SUMO laddering disappeared (supplemental Fig. S4*B*). This result indicated that the major SUMOylated lysine(s) remained to be identified.

We therefore employed MS/MS analysis to identify the remaining PYK2 SUMOylated lysine site(s). After WT PYK2 protein was immunoprecipitated from transfected cells (48 h post cotransfection with SUMO1 and PIAS1), mass spectrometry analysis identified a fourth lysine residue, K581, located within the kinase domain (data not shown). However, the K581R mutant remained strongly SUMOylated in the same cell-based validation assay (supplemental Fig. S4*C*). It was only when all four lysine residues were mutated to arginine (4KR) in a single construct that we observe a substantial reduction in target SUMOylation (Fig. 5*D*). Therefore, a 4KR mutant was used in the remainder of the study to characterize the impact of SUMOylation on PYK2's function.

##### SUMOylation Promotes PYK2 Autophosphorylation Activity

**Fig. 6.**
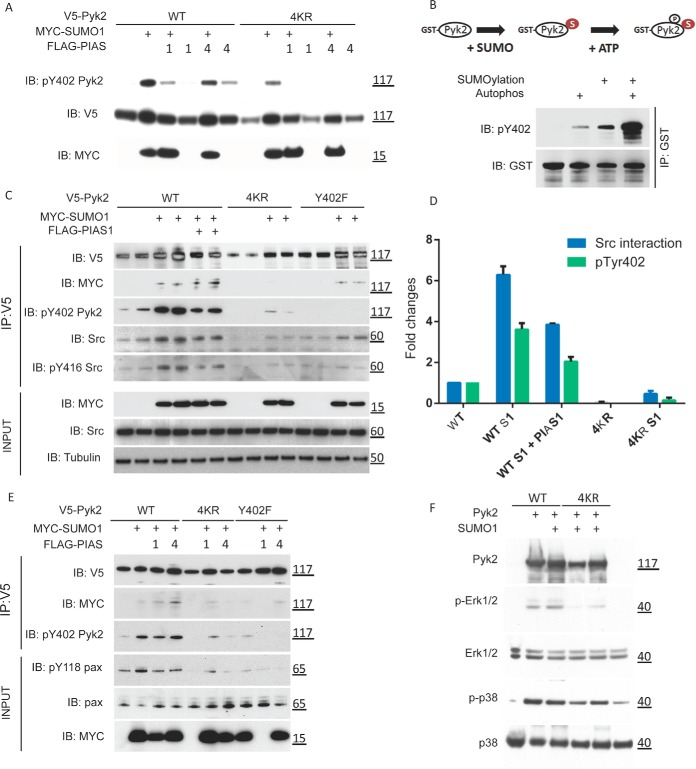
**PYK2 SUMOylation promotes its interaction with SRC.** *A*, Comparison of phosphorylation at Tyr402/579/580/881 in WT *versus* 4KR mutant. V5-PYK2 constructs were cotransfected with MYC-tagged SUMO1 along with FLAG-tagged PIAS1 or PIAS4 as indicated. Lysates were analyzed for phosphorylation status at Tyr402, Tyr579/80, and Tyr881 using phosphospecific antibodies. *B*, SUMOylation stimulates autophosphorylation of PYK2 *in vitro*. Recombinant GST-PYK2 was SUMOylated by SUMO1 in the absence of an E3 ligase. Autophosphorylation was examined for the following conditions: untreated, autophosphorylated (kinase assay), SUMOylated, SUMOylated then autophosphorylated (kinase assay). The samples were resolved by SDS-PAGE and subjected to Western blotting with anti-pTyr402 antibody. *C*, SUMOylation of PYK2 promotes interaction with SRC. HeLa cells were transfected with plasmids encoding for V5-tagged PYK2, V5-PYK2 containing four lysine to arginine mutations (4KR), and V5-PYK2 Y402F. V5-PYK2 constructs were cotransfected with MYC-tagged SUMO1 along with FLAG-tagged PIAS1. V5-PYK2 was immunoprecipitated under nondenaturing conditions to assess the interaction with endogenous SRC. Phosphorylation status of PYK2 and SRC were also analyzed using phosphospecific antibodies. Inputs controls for MYC-SUMO1, endogenous SRC and Tubulin are also shown. *D*, Quantitative analysis of PYK2 autophosphorylation and PYK2-SRC interaction based on biological duplicates in Fig. 6*C. E*, SUMOylation of PYK2 promotes phosphorylation of paxillin. HeLa cells were transfected with plasmids encoding for V5-tagged PYK2, V5-PYK2 4KR, or V5-PYK2 Y402F. V5-PYK2 constructs were cotransfected with MYC-tagged SUMO1 along with FLAG-tagged PIAS1. Phosphorylation status of PYK2 adaptor protein paxillin was determined by Western blotting for pTyr118. *F*, PYK2 4KR mutant inhibits activation of ERK1/2 but not P38/MAPK. HEK293 cells were transfected with either WT or 4KR versions of PYK2, plus and minus SUMO1 coexpression. Whole cell lysates were subject to Western blotting to probe for activation of ERK1/2 (pERK1/2), total ERK1/2, activated P38 MAPK (pP38/MAPK), and PYK2 expression.

Previous studies focused on PYK2 signaling have identified four tyrosine residues, namely Tyr402, Tyr579, Tyr580, and Tyr881, which are important for its full activation (36, 37). Upon activation of the PYK2 signaling pathway, Tyr402 undergoes autophosphorylation as the initial step to activate downstream signaling and recruits SRC kinase. PYK2 interacts with SRC's SH2 domain forming a complex that results in transphosphorylation at Tyr579/580/881 by SRC to fully activate PYK2 (36, 37). Because autophosphorylation at Tyr402 is an essential step in PYK2's activation, we asked whether SUMOylation affects PYK2's autophosphorylation at Tyr402. Using a phospho-Tyr402 specific antibody, we observed that cotransfection of WT PYK2 with SUMO1 alone, or in combination with either PIAS1 or PIAS4, resulted in enhancement of the autophosphorylation signal at Tyr402 when compared to transfection with WT PYK2-alone (Fig. 6*A*). On the other hand, when the 4KR PYK2 mutant was cotransfected under the same conditions, the Tyr402 autophosphorylation signal was either undetectable or dramatically reduced irrespective of SUMO1 and/or PIAS1/4 cotransfection, suggesting that SUMOylation of PYK2 stimulates autophosphorylation of Tyr402 *in cellulo* (Fig. 6*A*). Given that enhanced autophosphorylation of PYK2 can result from either increased phosphorylation or inhibition of dephosphorylation events, we evaluated whether SUMOylation of PYK2 could directly affect its autophosphorylation activity by using purified recombinant proteins in an *in vitro* assay system. Purified GST-tagged WT PYK2 was first SUMOylated under the 50X E1/E2 condition. After the SUMOylation machinery was washed away, GST-tagged protein was incubated in a standard kinase reaction buffer optimized for autophosphorylation. Phospho-Tyr402 signals were greatly enhanced when preceded by SUMOylation as compared with the PYK2 autophosphorylation without a prior SUMOylation reaction for WT PYK2 (Fig. 6*B*). To determine whether 4KR and K581R PYK2 mutants retain autophosphorylation activity *in vitro*, purified GST-tagged PYK2 lysine mutants were subject to autophosphorylation/SUMOylation assays. Importantly, the K581R and 4KR mutants demonstrated *in vitro* autophosphorylation activity, indicating neither loss of SUMOylation nor mutation of K581 could significantly affect its kinase activity (supplemental Fig. S5*A*). Together, these results demonstrate that SUMOylation directly increases the intrinsic autophosphorylation activity of PYK2, and that K581 is not required for autophosphorylation activity. However, we cannot entirely rule out that the lysine mutations, aside from reduction in SUMOylation, contribute to biological effects that we have not characterized.

##### SUMOylation Stimulates SRC Association with PYK2

Because it has been well established that Tyr402 autophosphorylation of PYK2 serves as the initial signal for recruiting SRC kinase via its SH2 domain, leading to subsequent activation (36), we performed IP-WB analysis to examine the role of SUMOylation on PYK2-dependent recruitment of SRC. Compared with expression of WT PYK2 alone, PYK2 coexpressed with SUMO1 or SUMO1/PIAS1 showed a greater ability to recruit SRC, in terms of both total SRC and its activated form (*i.e.* pTyr416 SRC), presumably because of enhanced pTyr402 PYK2 autophosphorylation (Fig. 6*C*). As expected, 4KR PYK2 expressed alone or with SUMO1 showed much reduced ability to recruit SRC (Fig. 6*C*). A surprising result was the observation that Tyr402F PYK2 -SUMO1 was able to rescue the association with SRC, despite the lack of autophosphorylation.

WT PYK2, 4KR, and a PYK2 Tyr402F mutant expressed with and without SUMO1, resulted in stimulation of SRC interaction with PYK2-SUMO1, but non-SUMOylated PYK2, 4KR, or 4KR coexpressed with SUMO1 did not (Fig. 6*C*). Because it was reported that SRC's recruitment to PYK2 was by direct binding to autophosphorylated Tyr402, we tested whether PYK2 SUMOylation was affected by autophosphorylation activity. The observation that the PYK2 Tyr402F became SUMOylated as efficiently as WT PYK2 (supplemental Fig. S5*B*) indicated that Tyr402 phosphorylation does not affect the SUMOylation status of PYK2. Surprisingly, SUMO1, when cotransfected with PYK2 Tyr402F construct, could partially rescue the association between SRC and PYK2 (Fig. 6*C*), despite the lack of detectable autophosphorylation. Therefore, PYK2-SRC interaction is not entirely dependent on Tyr402 phosphorylation but instead, can also be mediated through SUMOylation (Fig. 6*C*). Quantitative analysis further confirmed our observation (Fig. 6*D*).

It was well established that interaction between SRC and autophosphorylated PYK2 regulates SRC activity via phosphorylation at Tyr416 in SRC, which, in turn, fully activates PYK2 activity via tyrosine phosphorylation at other sites on PYK2 (36–38). We therefore performed IP-WB analysis to examine the SRC Tyr416 phosphorylation status under various conditions. As expected, Tyr416 of SRC was readily phosphorylated in cells only when WT PYK2 was SUMOylated following either cotransfection with SUMO1 or SUMO1/PIAS1; whereas SRC Tyr416 phosphorylation was almost undetectable in cells transfected with the PYK2 4KR mutant construct (Fig. 6*C*). Interestingly, though the PYK2 Tyr402F mutant was SUMOylated and could interact with SRC, it failed to stimulate Tyr416 phosphorylation in SRC (Fig. 6*C*). These observations strongly suggest that autophosphorylation at Tyr402 and SUMOylation at K35/145/581/895 in PYK2 synergistically enhance PYK2 and SRC interaction, serving as a positive feedback loop that is necessary to fully activate the SRC-PYK2 complex.

##### PYK2 SUMOylation Stimulates Phosphorylation of Downstream Signaling Molecules

As a focal adhesion protein, paxillin constitutively interacts with PYK2 and FAK, and is a known *in vivo* target of PYK2 phosphorylation. Therefore, we examined whether PYK2 SUMOylation also played a role in activating downstream targets of PYK2 involved in focal adhesion dynamics. Phosphorylation of paxillin at tyrosine 118 is associated with focal adhesion turnover (39). In a similar IP-WB analysis, we observed that tyrosine 118 phosphorylation of endogenous paxillin was strongest when WT PYK2 was SUMOylated (*i.e.* cotransfected with SUMO1) but diminished when PYK2 could not be SUMOylated (*i.e.* the 4KR mutant) or incapable of autophosphorylation (*i.e.* the Tyr402F mutant) (Fig. 6*E*). On the other hand, the overall paxillin protein level was not significantly affected by the status of PYK2 SUMOylation or Tyr402 autophosphorylation (Fig. 6*E*).

Paxillin plays an important role as a scaffold protein in MAPK pathway activation, critical for regulating cytoskeletal dynamics and cell migration (40). Based on our result that PYK2-SUMO1 stimulates phosphorylation of paxillin at Tyr118, which is within the Erk interaction site, we chose to examine the effect of PYK2-SUMOylation on Erk activation (40, 41). To better evaluate the role of SUMOylation within this signaling pathway, HEK293 cells were transfected with WT PYK2 and 4KR, with and without SUMO1 cotransfection, to examine downstream MAPK activation (Fig. 6*F*). In the presence of WT PYK2, increased phosphorylated ERK1/2 levels were observed in accordance with the trend we noted with paxillin phosphorylation. Also in line with our previous results, expression of the 4KR PYK2 impaired ERK1/2 phosphorylation, although coexpression of SUMO1 modestly stimulated phospho-ERK1/2 signal. Phosphorylation of p38 was enhanced in the presence of WT and 4KR PYK2, however, we did not observe a SUMO dependent increase in activation. These experiments reveal that disruption of SUMOylation by the 4KR mutant specifically impairs ERK1/2 activation.

##### PYK2 SUMOylation Promotes Cell Migration via Phosphorylation of Paxillin

**Fig. 7.**
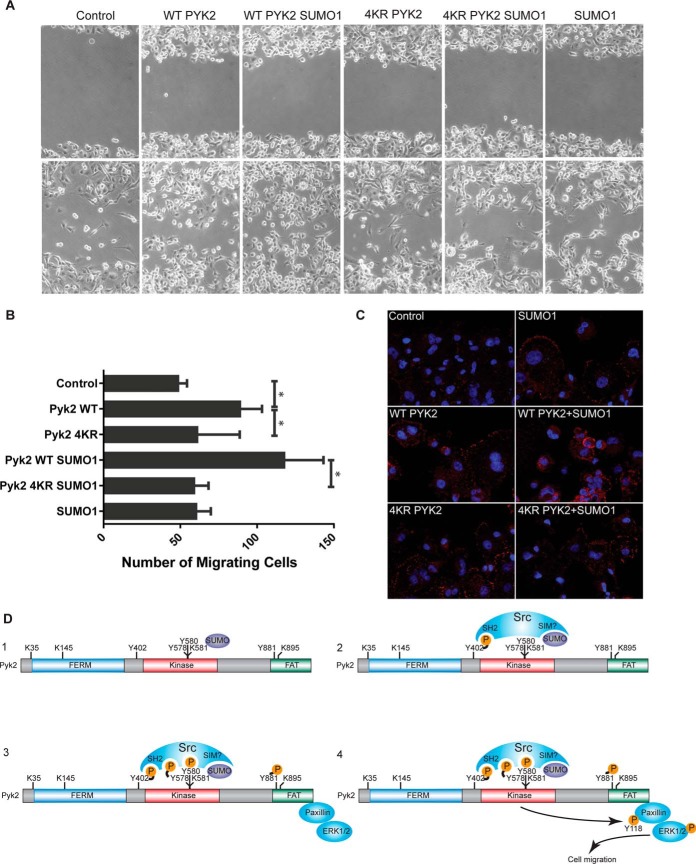
**PYK2 SUMOylation promotes cell migration.** *A*, 2D scratch assay. MDA-MB-231 breast cancer cells were infected with adenovirus constructs as indicated, at MOI 30. The monolayer was scratched 24 h following infection and cells were allowed to migrate into the wound for 24 h in low serum (0.1% FBS). The number of migratory cells was counted 24 h post scratch (*n* = 3 per condition). *B*, Quantification of cell migration. The number of migratory cells in the WT condition is significantly more than control and 4KR PYK2 (*p* < 0.029 and *p* < 0.036, respectively, two tailed, *t* test). WT PYK2 SUMO1 condition is significantly larger than 4KR PYK2 SUMO (*p* < 0.046, two tailed, *t* test). *C*, Paxillin activation under PYK2-SUMOylation and wounding. Cells were set up identical to panel *A*. 4 h post scratch, cells were fixed and immunofluorescence assays were performed to detect paxillin phosphorylatedTyr-118. Nuclei (Blue); p-Tyr-118 paxillin (Red). *D*, Crosstalk-mediated activation of PYK2 model. In step **1**, SUMOylation of PYK2 occurs at Lys581 as well as other acceptor lysine sites. SUMOylation triggers autophosphorylation at Y402, which stimulates interaction with SRC through its SH2 domain as well as potential SIM-mediated interactions (step **2**). SRC phosphorylates PYK2 at Tyr579, Tyr580, and Tyr881 resulting in full catalytic activity of PYK2 (step **3**). PYK2-SUMO1 phosphorylates focal adhesion protein paxillin at Tyr118 and activates ERK1/2. pTyr118 is linked to cell migration likely through activation of the MAP kinase pathway (step **4**). SUMOylation of PYK2 uncovers a novel crosstalk-mediated mechanism for kinase activation and function.

Paxillin serves to coordinate activation of ERK at sites of focal adhesion to potentiate cell migration (40, 42). Although SUMOylation has not previously been shown to be involved in cell migration, our observations that PYK2 SUMOylation triggers paxillin phosphorylation raised the possibility that SUMOylation of PYK2 is an upstream event, which promotes cell migration through phosphorylation of paxillin. To test this hypothesis, we performed a cell migration assay using MDA-MB-231 metastatic epithelial breast cancer cells. Adenovirus encoding WT PYK2 or 4KR PYK2 was coinfected with or without the SUMO1 construct in MDA-MB-231cells. A confluent monolayer of cells expressing these constructs was wounded and cell migration was monitored over the next 24 h. As compared with the mock control and 4KR PYK2, expression of WT PYK2 stimulated cell migration into the wound, as expected (*p* < 0.029, *p* < 0.036, respectively; two lower left panels; Fig. 7*A*). Importantly, the number of migratory cells in the WT PYK2 SUMO1 coinfected cells was the highest and significantly larger (*p* < 0.046) than the 4KR PYK2 SUMO1infected cells (the 3^rd^ and 5^th^ lower panels from left; Fig. 7*A*). Consistently, cells infected with 4KR or SUMO1 constructs alone did not show any significant difference with the un-infected cells (Fig. 7*A*).

To assess whether migration stimulated by PYK2 SUMOylation triggers signaling through the focal adhesion pathway, paxillin pTyr118 expression, at 4 h post wounding, was monitored with a phosphospecific antibody (pTyr118) by immunofluorescence staining. Coexpression of WT PYK2 with SUMO1 showed augmented pTyr118 expression over all other conditions (Fig. 7*C*), consistent with our biochemical observations (Fig. 6*E*). WT PYK2, 4KR PYK2, 4KR PYK2 SUMO1 showed similar levels of pTyr118 paxillin staining, indicating that SUMO1 stimulates phosphorylation of paxillin through WT PYK2 to regulate cell migration. Similar levels of pTyr118 paxillin staining among WT PYK2, 4KR PYK2, 4KR PYK2 SUMO1 also suggests that the 4KR mutant is not intrinsically less active than the WT.

## DISCUSSION

In the past decade several large-scale studies have aimed to characterize the full collection of proteins that comprise the SUMOylome by employing MS/MS to identify SUMOylation substrates and sites (10, 12, 13, 29, 43, 44). Although these studies have provided us with a more detailed picture of the cellular SUMOylation landscape, the absence of E3 ligases in these data sets has hindered further understanding of the molecular and functional mechanisms regulating SUMOylation. Proteomic strategies that interrogate E3 ligase function, substrate identification, and SUMOylation conducted solely using IP/MS are inherently limiting. By using recombinant SUMO E3 ligases in conjunction with the richness of the HuProt™ arrays, our study has allowed a global profile of SUMO substrate-E3 ligase specificity to be generated and has revealed previously unappreciated cellular processes that are likely to be regulated via SUMOylation. Our direct *in vitro* analysis has yielded a comprehensive data set that integrates both E3 ligase and SUMO isoform specificity, enabling us to define the role of E3 ligases in substrate selection and SUMO isoform preference at a proteomic level. In this report, we identify 3640 SUMOylation substrates (50X + All E3s) of which 2150 were E3 ligase targets. This represents the first effort to globally characterize E3 ligase specificity. Of note, our study identified 118 (19.9%), 48 (19.1%), 1445 (36.7%) of the SUMOylated proteins reported by Tammsalu *et al.* 2014, Impens *et al.* 2014, and Hendriks *et al.* 2017, respectively. These and other proteomic studies employ cell lines transfected with tagged SUMO expression vectors, often subjected to cellular stress, followed by affinity purification of SUMOylated proteins coupled to MS/MS analysis. With this specialized system, efficiently modified, high abundance substrates are readily identified irrespective of E3 ligase activity. Most of the substrates identified by Tammsalu and Impens were SUMOylated within the consensus site that is known to be SUMOylated via Ubc9-directed SUMOylation, presumably because of Ubc9's ability to directly bind to the consensus motif (13, 44–46). Although it is encouraging that our findings do overlap with the results from these MS-based studies, we suspect that the proteins modified in the protein microarray screen represent a distinct set of substrates that may require specific biochemical conditions to undergo modification, or else be expressed at low levels in the cell lines tested in these studies. Furthermore, as previously discussed, the majority of E3 ligase-specific targets we report did not contain consensus motifs, supporting the theory that E3 ligase-mediated SUMOylation largely regulates modification of substrates lacking the classical consensus SUMOylation site, which affinity purification-MS/MS screens may not be optimized to detect.

Many scientists in the field have used hypothetical E3 ligase function to explain how SUMOylation substrate specificity is controlled; however, this theory was not supported by the small number of empirical studies linking E3 ligases to SUMOylation of specific substrates (47, 48). Here, we were able to generate the largest data set to explain the numerous roles that E3 ligases play in regulating SUMO substrate selection. Indeed, this study revealed E3 ligase specific substrates (*e.g.* those only modified in the presence of a particular E3 ligase), substrates that are shared or redundant (*e.g.* those modified in more than one condition), and those that do not show E3 dependence (*e.g.* those only modified under high E1 and E2 conditions). Even superficially, we can note vivid distinctions in the E3 ligase properties. Perhaps, the most obvious is the great variation in the number of substrates that each E3 modified in conjunction with different SUMO isoforms. A unifying feature is that they each modify a subset of substrates specifically. Although the literature indicates instances where E3 ligase demonstrate SUMO isoform specificity (*e.g.* PIAS4 preference for SUMO2) (48), we were surprised by the dramatic SUMO isoform preference exhibited by PIAS3, PIAS4, and TOPORS. The molecular mechanism behind this observation will require further investigation.

SUMO isoforms have been shown to have roles in different biological processes and thus, we expected that the global level of overlap in substrates modified by SUMO1 and SUMO2 would be relatively modest. It has long been speculated that E3 ligases are responsible for directing SUMOylation specificity in two capacities: by selecting the substrate and by discriminating between SUMO isoforms (1, 9). Our observation that many substrates that were only modified by one SUMO isoform under the 50X E1/E2 condition could be readily modified by SUMO1 and SUMO2 in the presence of an E3 ligase, suggests that E3 ligases may mediate selective attachment of individual SUMO isoforms to hundreds of different proteins.

SUMO E3 ligases coordinate modification of specific substrates presumably for explicit biological purposes. Our GO analysis revealed enrichment in previously reported categories, such as DNA damage, protein transport, transcription regulation, and stress response, as well as many novel biological processes and molecular functions. These include small GTPase signaling, phosphorylation, ligase activity, Wnt receptor signaling, and protein folding - all currently unexplored areas for SUMOylation function. In combination with the GO results from other SUMO proteomics studies we are now building an extensive global map of cellular processes where SUMOylation is critical. Enrichment for phosphorylation and kinase activity suggests possible systems level connections between phosphorylation and SUMOylation. Individual studies of several substrates suggest that crosstalk between SUMOylation and phosphorylation may coregulate protein function (49–53). This phenomenon is further supported by a large-scale study of SUMO-regulated phosphorylation wherein the authors report that expression of SUMO2/3 in HEK293 cells stimulates an increase in global tyrosine phosphorylation (54). Comodification of SUMOylation and phosphorylation was also a major finding in the large scale study MS/MS study which shares the largest overlap of targets with our data set (29).

Our characterization of the function of PYK2 SUMOylation exemplifies this earlier observation, linking SUMOylation and enhanced global tyrosine phosphorylation. PYK2 autophosphorylation is well characterized in the context of integrin signaling, G protein activation, and calcium signaling; however, the accepted mechanism described trans-autophosphorylation at pTyr402 as the key PTM controlling this process (36, 55). Our study has revealed a novel paradigm for the activation mechanism of PYK2 in which SUMOylation enhanced autophosphorylation of PYK2, in the absence of an upstream stimulus. We found that the role of SUMOylation extended beyond intramolecular activity, as SUMOylation of PYK2 kinase-dead mutant (Tyr402F) was able to recruit bona fide interaction partner SRC, which was presumed to interact with autophosphorylated PYK2 only through its SH2 domain. This interaction cannot produce full activation of the enzymes. Our findings thus illustrate a new mechanism where the two PTMs cooperate to generate full activation of the PYK2 at Tyr402, Tyr579, Tyr580, and Tyr881.

In this study, we have provided evidence that SUMOylated PYK2 enhances motility of MDA-MB-231 metastatic breast cancer cells via signaling through the SRC, paxillin, and ERK1/2 signaling cascade. In the context of our results, overexpression of PYK2 promotes cell migration, and coexpression with SUMO augments the migration phenotype. Identifying the endogenous dynamics of PYK2 SUMOylation is critical for understanding the importance of this finding. Likely, PIAS1 or PIAS4 mediates SUMO modification of PYK2 *in vivo*, stimulating its autophosphorylation, association with SRC, and phosphorylation of paxillin to elicit cell migration. Based on our collective results, we propose a crosstalk-mediated signaling cascade whereby SUMOylation of PYK2 stimulates its autophosphorylation activity, interaction with SRC, paxillin phosphorylation, and ERK activation resulting in initiation of cell migration pathways (Fig. 7*C*).

It is possible that SUMO plays a broader role in cell migration than simply mediating PYK2 dynamics. Rac1 is a member of the Rho GTPase family that is known to regulate cell migration, adhesion dynamics, and cytoskeleton remodeling (56, 57). SUMOylation was demonstrated to function in cell migration by modifying and activating GTPase Rac1 in MEF cells (56). PIAS3 was identified as an E3 ligase for RAC1, and down-regulation of PIAS3 resulted in impaired migration compared with controls. Phosphorylation of p38, which is a known downstream mediator of Rac1 signaling, as well as a MAPK SUMOylation substrate in our assays, was also impaired in PIAS3-downregulated cells (56).

Our studies illustrate so-called “higher-order signaling machines” which rely on proximity driven enzyme activation to generate signal amplification and possibly temporal spatial regulation of signal transduction (58). Broadly, we have illuminated the connections between SUMOylated kinases along a signaling axis, promoting enzyme activity, protein interactions, and activation of numerous nodes in a pathway. More specifically, characterization of PYK2 SUMOylation describes a novel mechanism wherein SUMO modification drives amplification of autophosphorylation, interaction with SRC, phosphorylation of paxillin, activation of ERK1/2, and cell migration.

## DATA AVAILABILITY

All data sets generated and referenced in this study have been supplied as supplementary files.

## Supplementary Material

Supplemental Data

## Footnotes

^1^ The abbreviations used are:

PTM: post translational modification
SUMO: small ubiquitin-related modifier
MAPK: mitogen-activated protein kinase
GST: glutathione *S*-transferase
PPI: protein-protein interaction
RIPA: radioimmunoprecipitation assay buffer
IP: immunoprecipitation
IB: immunoblot
PIAS: protein inhibitor of activated STAT
GO: gene ontology
KSR: kinase substrate relationship
ORF: open reading frame
WT: wild type.
