# Supplementary material for: Global Identification of Small Ubiquitin-related Modifier (SUMO) Substrates Reveals Crosstalk between SUMOylation and Phosphorylation Promotes Cell Migration
Source: Mol Cell Proteomics. 2018 Feb 8;17(5):871–88. doi: 10.1074/mcp.RA117.000014 (PMC5930406; doi:10.1074/mcp.RA117.000014)
Supplement: Supplemental Data [file supp_17_5_871__index.html]

Global Identification of Small Ubiquitin-related Modifier (SUMO) Substrates Reveals Crosstalk between SUMOylation and Phosphorylation Promotes Cell Migration — Global SUMOylation Study Reveals PTM Crosstalk — Supplemental Data 

# Global Identification of Small Ubiquitin-related Modifier (SUMO) Substrates Reveals Crosstalk between SUMOylation and Phosphorylation Promotes Cell Migration

## Supplemental Data

- Supplemental Figure 1 - A boutique protein microarray containing 82 GST-tagged SUMO substrates and conjugation enzymes printed in duplicate.
- Supplemental Figure 2 - Activity of purified E3 ligases on reported substrates
- Supplemental Figure 3 -

  Reproducibility of protein microarray assay replicates
- Supplemental Figure 4 - GO compartmental analysis and Identification of SUMO1 modified lysine residues in PYK2
- Supplemental Figure 5 - Comparison of autophosphorylation Pyk2 and mutants
- Supplemental Figure Legends - Supplemental Figure legends
- All SUMOylated proteins - All proteins that were SUMOylated, included those modified in the absence of an E3 ligase
- Motif site analysis - SUMOylation occurring in consensus motif with M3 and standard alogorithms.
- Table of All hits combined - Listing of all proteins on protein microarray that were modified, under specific conditions, in protein array screening assay
- Mass Spec Data Explanation - Explanation for unavailable mass spec data
