# Supplementary material for: Global Identification of Small Ubiquitin-related Modifier (SUMO) Substrates Reveals Crosstalk between SUMOylation and Phosphorylation Promotes Cell Migration
Source: Mol Cell Proteomics. 2018 Feb 8;17(5):871–88. doi: 10.1074/mcp.RA117.000014 (PMC5930406; doi:10.1074/mcp.RA117.000014)
Supplement: Supplemental Data [file supp_RA117.000014_4537_2_supp_64132_p3r48r.pdf]

Supplemental Figure 1

Pilot Array SUMOylation

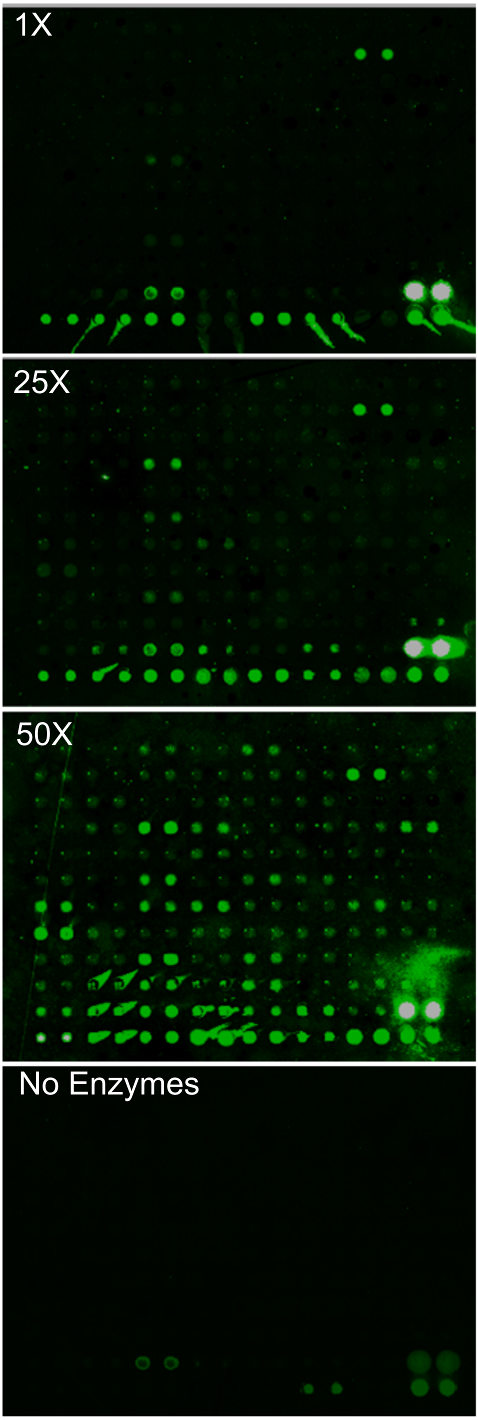

Anti-SUMO1 primary antibody
Alexa555-g secondary antibody
