# Supplementary figures and images for: Global Identification of Small Ubiquitin-related Modifier (SUMO) Substrates Reveals Crosstalk between SUMOylation and Phosphorylation Promotes Cell Migration
Source: Mol Cell Proteomics. 2018 Feb 8;17(5):871–88. doi: 10.1074/mcp.RA117.000014 (PMC5930406; doi:10.1074/mcp.RA117.000014)

Supplemental Figure 2

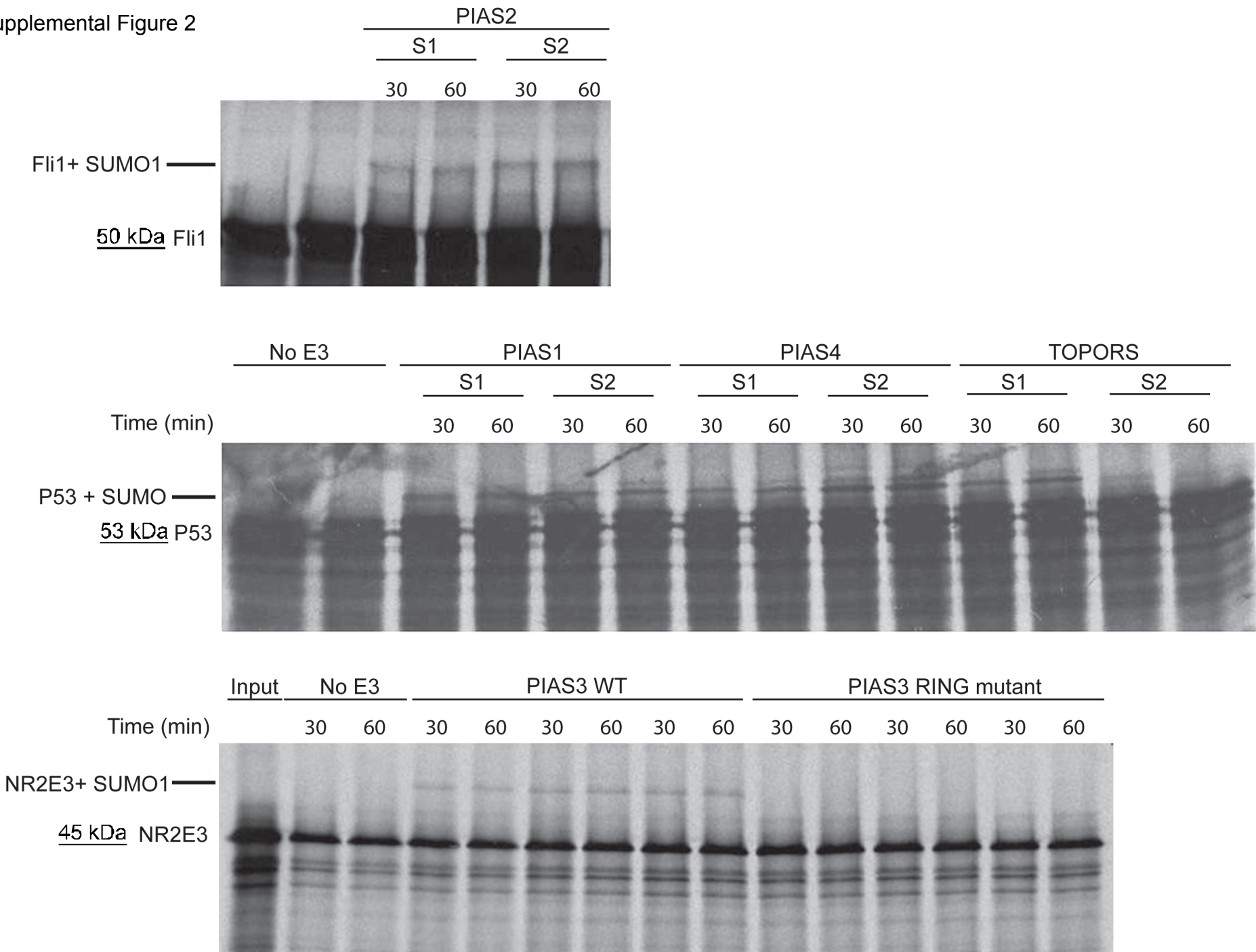

Supplement: Supplemental Data [file supp_RA117.000014_4537_2_supp_64133_p3r18s.pdf]

A

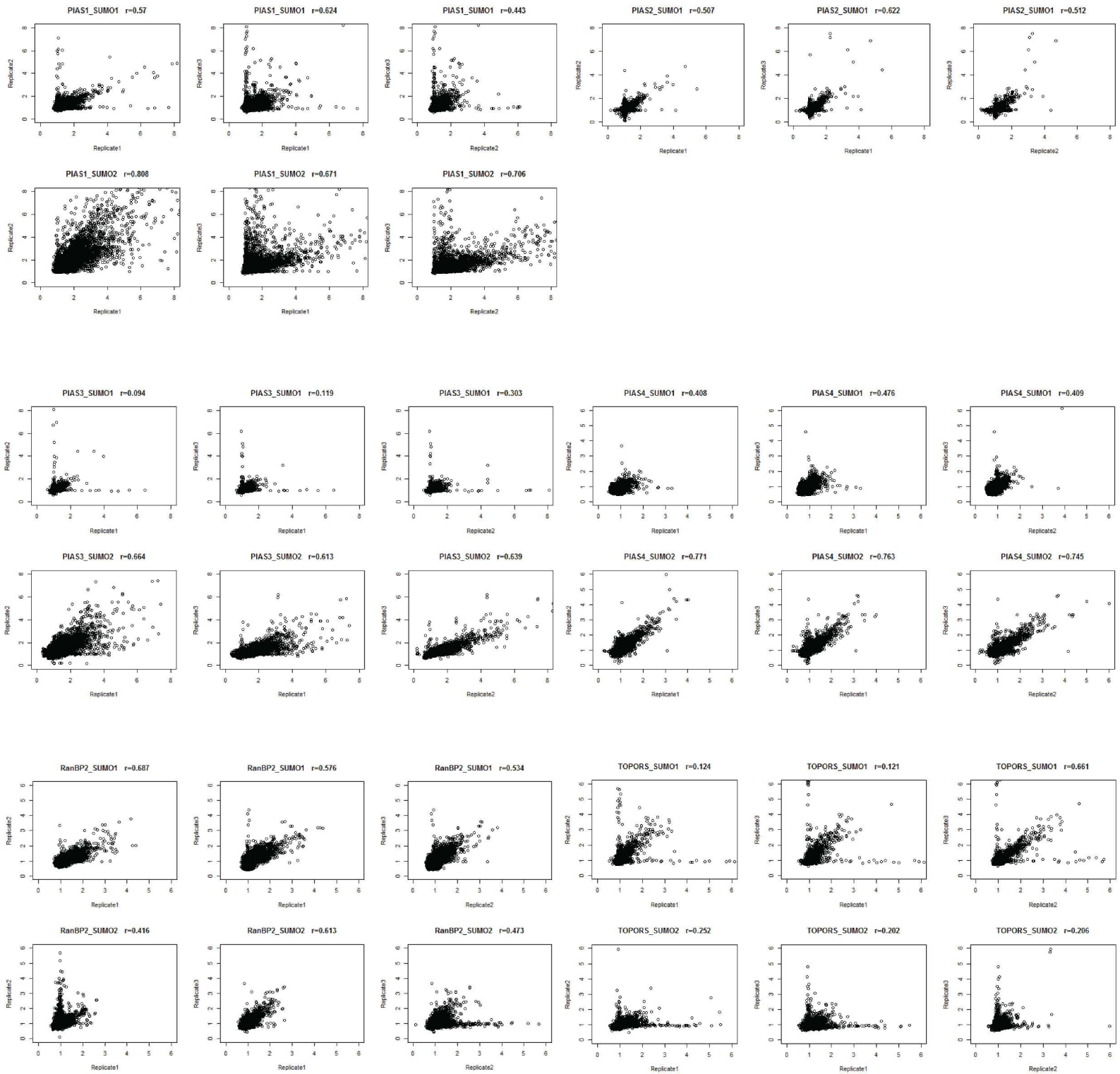

Supplement: Supplemental Data [file supp_RA117.000014_4537_2_supp_64134_p3r78r.pdf]

A

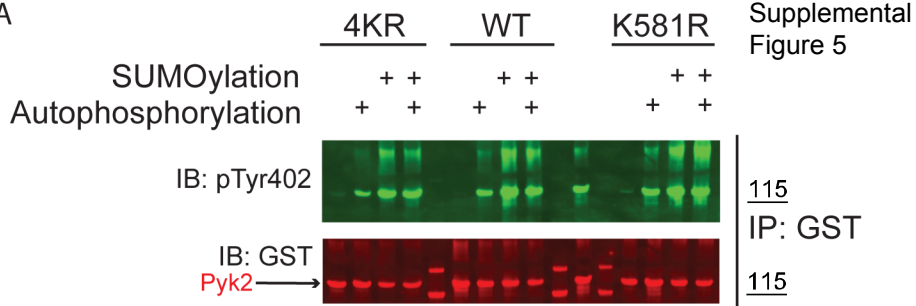

B

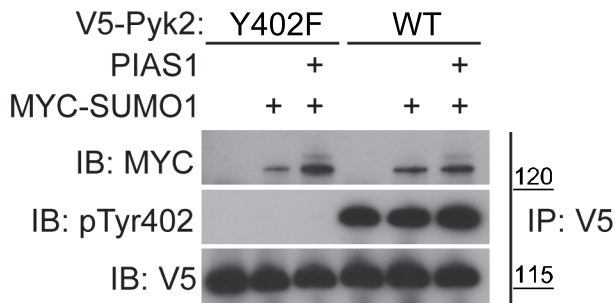

Supplement: Supplemental Data [file supp_RA117.000014_4537_2_supp_64139_p3rp8r.pdf]
