# Supplementary material for: Global Identification of Small Ubiquitin-related Modifier (SUMO) Substrates Reveals Crosstalk between SUMOylation and Phosphorylation Promotes Cell Migration
Source: Mol Cell Proteomics. 2018 Feb 8;17(5):871–88. doi: 10.1074/mcp.RA117.000014 (PMC5930406; doi:10.1074/mcp.RA117.000014)

Supplemental Figure 1

A

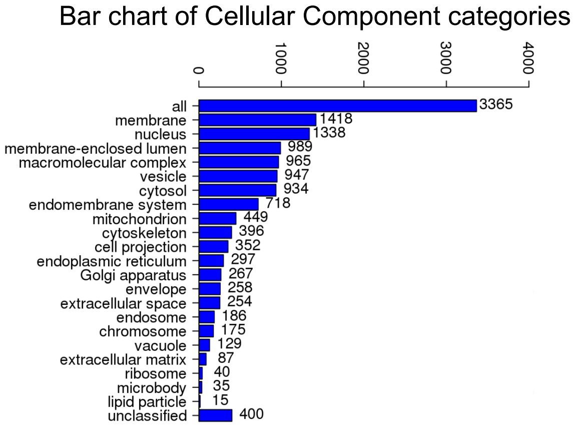

Term	GO ID	#Genes in list	#Expected	Enrichment	P value	FDR
Nucleus	GO:0005634	1338	520	2.57	0	0

B

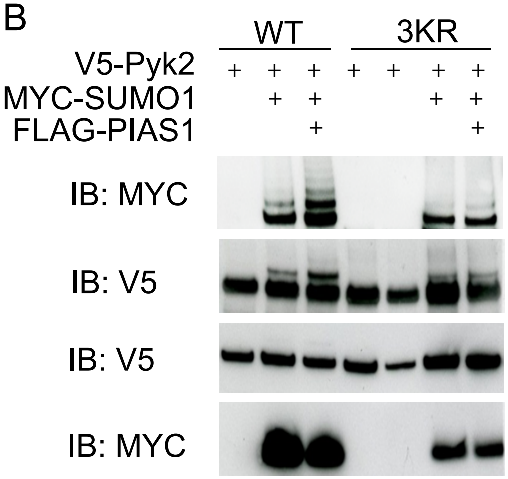

C

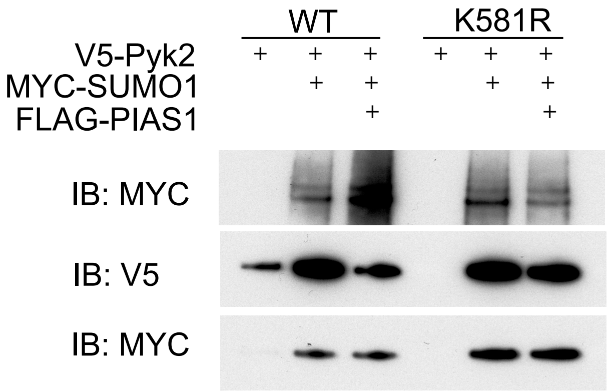

Supplement: Supplemental Data [file supp_RA117.000014_4537_2_supp_64141_p3rs8s.pdf]
