# Supplementary material for: Global Identification of Small Ubiquitin-related Modifier (SUMO) Substrates Reveals Crosstalk between SUMOylation and Phosphorylation Promotes Cell Migration
Source: Mol Cell Proteomics. 2018 Feb 8;17(5):871–88. doi: 10.1074/mcp.RA117.000014 (PMC5930406; doi:10.1074/mcp.RA117.000014)
Supplement: Supplemental Data [file supp_RA117.000014_4537_2_supp_64131_p3k5lj.pdf]

Figure S1 (related to Figure 1)

A boutique protein microarray containing 82 GST-tagged SUMO substrates and conjugation enzymes printed in duplicate. The pilot chip was incubated with increasing concentrations of E1 and E2 enzymes (1X, 25X, 50X) to determine the appropriate concentration for high and low concentration controls for the HuProt™ global screening assays. Covalently attached SUMO1 is detected with anti-SUMO1 (21C7) primary antibody and Alexa 555 secondary antibody.

Figure S2 (related to Figure 1)

Activity of purified E3 ligases on reported substrates: PIAS1/Fli1; PIAS1,PIAS4,TOPORS/P53;PIAS3, PIAS3 RING mutant/NR2E3. [^{35}S] methionine-labeled substrates were transcribed and translated in rabbit reticuloctye lysate. Substrates were SUMO modified with limiting concentrations of E1 and E2 with and without indicated E3 ligases to confirm activity *in vitro*. Samples were collected at 30 and 60 min, resolved on SDS pages gels, and visualized by autoradiography.

Figure S3

A) HuProt™ array SUMOylation reproducibility. Pairwise Pearson correlation coefficients computed for three technical replicates for HuProt™ SUMOylation reactions containing E3 ligase enzymes.

B) HuProt images corresponding to IP Western blot results from Figure 4C.

Figure S4. Identification of SUMO1 modified lysine residues in PYK2 (related to Figure 5)

A) Cellular component analysis of full dataset, demonstrating 1338 nuclear proteins, representing 2.57 fold enrichment over the expected number (520 proteins)(P value = 0); WebGestalt 2017.

B) SUMOylation of V5 constructs of 3KR mutant (K35R, K145R, K895R) PYK2 mutant and WT PYK2 were transfected into HeLa cells. In parallel, mutant and WT kinase were co-transfected with SUMO1, and separately co-transfected with SUMO1 and PIAS1. V5-tagged

substrates were immunoprecipitated, resolved by SDS-PAGE, then blotted with anti-MYC to detect SUMOylation.

C) SUMOylation of K581R PYK2 mutant and WT PYK2. Mutant and WT V5 kinase constructs were co-transfected with SUMO1, and separately co-transfected with SUMO1 and PIAS1. V5-tagged substrates were immunoprecipitated, resolved by SDS-PAGE, then blotted with anti-MYC to detect SUMOylation.

Figure S5 (related to Figure 6)

A) Recombinant GST tagged WT PYK2, Y402F, and 4KR were subject to different permutations of SUMOylation or autophosphorylation to evaluate activation of PYK2 at Y402 as indicated in each lane. Autophosphorylation (phospho-Y402 signal) was examined for the following conditions: untreated, autophosphorylated (kinase assay), SUMOylated, SUMOylated then autophosphorylated (kinase assay). The samples were resolved by SDS-PAGE and subjected to western blotting with anti-pTyr402 and anti-GST antibodies.

B) Autophosphorylation is not required for SUMOylation of PYK2. SUMOylation of autophosphorylation deficient PYK2 mutant Y402F and WT PYK2. Y402F Mutant and WT V5 kinase constructs were co-transfected with SUMO1, and separately co-transfected with SUMO1 and PIAS1. V5-tagged substrates were immunoprecipitated, resolved by SDS-PAGE, then blotted with anti-MYC to detect SUMOylation and anti- pTyr402 to detect autophosphorylation activity.
