# Supplementary material for: Global Identification of Small Ubiquitin-related Modifier (SUMO) Substrates Reveals Crosstalk between SUMOylation and Phosphorylation Promotes Cell Migration
Source: Mol Cell Proteomics. 2018 Feb 8;17(5):871–88. doi: 10.1074/mcp.RA117.000014 (PMC5930406; doi:10.1074/mcp.RA117.000014)
Supplement: Supplemental Data [file supp_RA117.000014_4537_2_supp_68197_p3k5lj.pdf]

Department of Pharmacology
and Molecular Sciences
733 North Broadway / BRB 333
Baltimore, Maryland 21205
Phone: 410-502-0878
hzhu4@jhmi.edu
www.hopkinsmedicine.org/pharmacology/

JOHNS HOPKINS
MEDICINE

February 1, 2018

Dr. Pierre Thibault
Associate Editor
Molecular & Cellular Proteomics

Dear Dr. Thibault

We regret to say that the original MS/MS data were lost due an accident that happened in Dr. Hui Zhang's lab. Unfortunately, the postdoc fellow, Dr. Jianying Zhou, who did the MS/MS analysis, has left the Zhang lab a while ago. This was the reason that we did not add the MS/MS data in our previous submission. However, we did have solid mutagenesis studies and in vitro biochemistry evidence to demonstrate that SUMOylation did happen at the four lysine residues, including the one identified with MS/MS analysis (see Figures 5C, 5D, Supplemental Figure 4A). Therefore, we believe that the MS result was trustworthy.

Best regards,

Heng Zhu, Ph.D.
Professor of Pharmacology & Molecular Sciences
